# Direct and indirect effects of the COVID-19 pandemic on mortality: an individual-level population-scale analysis.

**DOI:** 10.23889/ijpds.v7i3.1922

**Published:** 2022-08-25

**Authors:** Rhiannon Owen, Jane Lyons, Ashley Akbari, Gareth Davies, Rowena Griffths, Fatemeh Torabi, Ronan Lyons

**Affiliations:** 1 Swansea University

## Objectives

The COVID-19 pandemic has had a detrimental impact on healthcare utilisation, resulting in increased mortality both directly and indirectly associated with COVID-19. We aimed to assess the impact of the COVID-19 pandemic on all-cause and disease-specific mortality and further explore the impact of potential inequalities, deprivation status and ethnicity.

## Approach

Population-scale, individual-level, anonymised linked, routinely-collected electronic health records from demographic and administrative sources were used for two cohorts: i) C19-COHORT16 included individuals alive and resident in Wales on the 1st January 2016 with follow-up until death, break-in Welsh residency, or 31st December 2019; ii) C19-COHORT20 included individuals alive and resident in Wales on 1st January 2020 with follow-up until death, break-in Welsh residency, or study end. We used time-series analysis to investigate trends in mortality over time. We fitted negative binomial models to estimate expected all-cause and disease-specific mortality and compared these estimates to observed mortality in C19-COHORT20.

## Results

Excess all-cause and COVID19-related deaths were higher during the period where the alpha variant was dominant. The trend in deaths decreased during the omicron dominant period. The Asian population had increased mortality during the period where the delta variant was dominant. Mortality was increased for most deprived groups compared to least deprived groups, however, the magnitude of this effect remained unchanged during the pandemic. COVID-19 indirectly affected cancer, circulatory, trauma, digestive and mental health related deaths, with a higher than expected mortality. The majority of trauma related deaths occurred early on in the pandemic, where a higher than expected number of deaths occurred outside of an NHS establishment. Mortality associated with respiratory disease (unrelated to COVID-19) was significantly lower than expected during the COVID-19 pandemic.

## Conclusion

Increased all-cause and disease-specific mortality was observed during the COVID-19 pandemic. Excess deaths may be a result of reduced healthcare utilisation, delayed investigation and/or treatment of chronic diseases. As healthcare systems recover from COVID-19, investigation of mortality trends will play a central role in healthcare planning, utilisation and resource use.

